# Stroke-like-migraine-attacks-after-radiation-therapy(SMART)-Syndrom: eine seltene Folge nach zerebraler Strahlentherapie

**DOI:** 10.1007/s00115-022-01413-z

**Published:** 2022-12-09

**Authors:** Stephanie Straub, Eva Bürkle, Alexander Grimm

**Affiliations:** 1 Abteilung Neurologie mit Schwerpunkt Epileptologie, Universitätsklinik Tübingen, Hoppe-Seyler-Str. 3, 72076 Tübingen, Deutschland; 2 Diagnostische und Interventionelle Neuroradiologie, Universitätsklinikum Tübingen, Tübingen, Deutschland Hoppe-Seyler Str. 3,

## Anamnese und klinischer Befund

Wir berichten über eine 51-jährige Patientin, die uns aufgrund einer seit mehreren Wochen bestehenden und subakut aufgetretenen Gedächtnis‑, Sprach- und Sehstörung unter der Verdachtsdiagnose einer zerebralen Vaskulitis vorgestellt wurde. Anamnestisch wurde einerseits von Gesichtsfeldeinschränkungen und einer verringerten Sehschärfe seit mehreren Wochen berichtet. Zudem bestanden anhaltende Wortfindungsstörungen, Orientierungsschwierigkeiten (zu Ort und Zeit) und ein streng linksseitiger Kopfschmerz von stechendem Charakter mit hoher Intensität, begleitet von Übelkeit und einem Ruhebedürfnis. Die Charakteristik des Kopfschmerzes konnte als Migräne ohne Aura (International Classification Of Headache Disorders 3 [ICHD 3]: 1.1) klassifiziert werden.

Die zeitliche Versetzung der Symptomatik in Kombination mit dem Nachweis teils diffusionsgestörter Areale in der MRT ließ die Annahme mehrzeitiger Ischämien zu. Aufgrund dessen und bei sonst fehlenden kardiovaskulären Risikofaktoren kam zunächst eine vaskulitische Genese in Betracht. Residuell bestand eine rechtsseitige, rein motorische und spastische Hemiparese nach kompletter Resektion eines Ependymoms (WHO Grad III) im Bereich des 4. Ventrikels linksseitig mit anschließender Ganzhirnbestrahlung (Strahlendosis unbekannt) im Dezember 1996. Für diese bestand aktuell keine Veränderung. Aus früheren MR-Aufnahmen war bereits eine strahleninduzierte Leukenzephalopathie mit multiplen Marklagerläsionen bekannt. Klinisch-neurologisch zeigten sich aktuell kognitive Defizite für komplexere Handlungen, eine expressive Aphasie sowie eine homonyme Hemianopsie nach rechts. Es bestand kein Meningismus und keine erhöhten Körpertemperaturen. Die Entzündungs- und Basislaborparameter sowie der CRP-Wert (< 0.05 g/l) waren bei Aufnahme normwertig. Hinweise auf eine endokrine oder metabolische Störung (unauffällige Schilddrüsenhormone und -antikörper, Nieren- und Leberwerte) ergaben sich nicht. Medikamente wurden bis auf den bedarfsgerechten Einsatz von Schmerzmitteln nicht eingenommen.

## Diagnose

### Bildgebung

**Figure 1:**
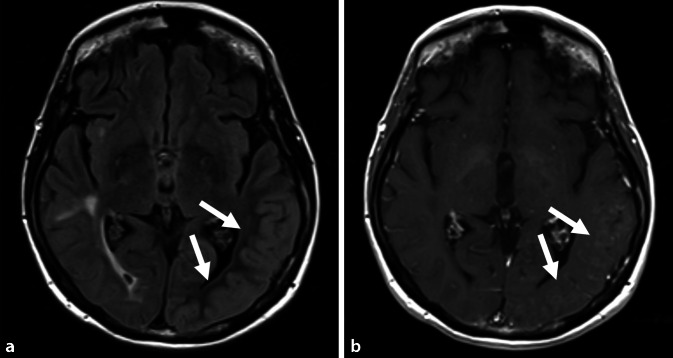

**Figure 2:**
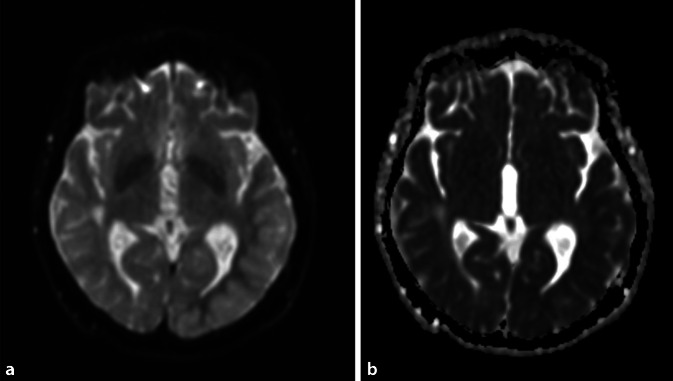

In Abb. 1 (a FLAIR axial, b kontrastangehobene T1-gewichtete Sequenz) ist jeweils der Zeitpunkt der akuten Symptomatik bestehend aus einer Gesichtsfeldeinschränkung, Wortfindungsstörungen, Orientierungsschwierigkeiten und den Kopfschmerzen erfasst. Hierbei sind deutlich eine Schwellung sowie Schrankenstörung der betroffenen Hirnareale (siehe weiße Pfeile) im Sinne einer Kontrastmittelaufnahme links temporal dargestellt. In der Diffusionswichtung ergaben sich entsprechend geringe kortikale Signalsteigerungen mit korrelierenden Signalabsenkungen in der „apparent diffusion coefficient map“ (ADC; Abb. 2). Gefäßirregularitäten zeigten sich in der CT-Angiographie und MR-TOF („time of flight“-Sequenz) nicht.

### Erweiterte Diagnostik

Unter Annahme eines infektiösen, autoimmun-entzündlichen oder vaskulitischen Geschehens erfolgte die Lumbalpunktion und ausführliche Labordiagnostik. Die Liquorzellzahl sowie das Laktat waren normwertig (Zellzahl 3/µl, Laktat 1,4 mmol/l). Bis auf ein leicht erhöhtes Liquoreiweiß mit 67 mg/dl zeigten sich keine Auffälligkeiten, unter anderem waren antineuronale Antikörper (Anti-Hu, Anti-Ri, Anti-Yo, Anto-Ma, Anti-GAD 65, Anti-NMDA-Rezeptor, Anti-LGI1, Anti-CASPR2, Anti-Zic4, Anti-SOX1), oligoklonale Banden und die vaskulitisassoziierten Antikörper (ANA, cANCA, pANCA, MPO-Ak und PR3-Ak) negativ. In der Dopplersonographie der Halsgefäße und der CT-Angiographie ergaben sich keine Hinweise auf eine Gefäßstenose oder -entzündung, in einer transösophagealen Echokardiographie waren ferner keine kardialen Pathologien darstellbar. Das Elektroenzephalogramm (EEG) ergab insgesamt niedrige Amplituden sowie eine milde fokale Hirnfunktionsstörung im Sinne einer intermittierenden Verlangsamung (5–6 Hz) rechts temporal, ohne Nachweis epilepsietypischer Potenziale, passend zur Lokalisation der vorbestehenden Leukenzephalopathie.

### Diagnosestellung

Nach genanntem Ausschluss wichtiger Differenzialdiagnosen konnte anhand der charakteristischen MRT-Bilder bestehend aus einseitigen, rein kortikalen Ödemen die Diagnose einer seltenen Strahlenfolge, dem SMART-Syndrom, gestellt werden. Dazu passten auch klinisch der subakute Beginn, die Sehstörung sowie der anhaltende Migränekopfschmerz und die Aphasie.

## Therapie und Verlauf

Nachdem die Diagnose eines SMART-Syndroms bildgebend und klinisch gestellt werden konnte, wurden 500 mg Methylprednisolon i.v. über 5 Tage verabreicht. Im Anschluss sollte die Kortisontherapie im Ausschleichschema (Startdosis Prednisolon 100 mg, jede Woche um 20 mg reduzieren) fortgeführt werden. Es folgte die stationäre Rehabilitationsbehandlung. Die Kopfschmerzen waren bereits bei Entlassung vollständig rückläufig, die Aphasie hatte sich dahingehend gebessert, dass zumindest ein flüssiges Gespräch in wenigen Worten möglich war. Die Patientin konnte nach 6 Tagen entlassen werden, Nebenwirkungen der begonnenen Medikation traten nicht auf. Im weiteren Verlauf über 3 Monate zeigten sich die Orientierungsstörung und Aphasie vollständig regredient, einzig die Gesichtsfeldeinschränkung war persistierend. Bildgebend zeigte sich im MRT nach 3 Monaten ein Rückgang des Ödems kortikal okzipital links sowie eine vollständige Regredienz der Schrankenstörung.

## Diskussion

Das SMART-Syndrom ist eine sehr seltene postradiogene Komplikation, die sich Jahre bis Jahrzehnte (1–30 Jahre) nach einer zerebralen Bestrahlung durch eine akut bis subakut auftretenden Fokalneurologie bemerkbar macht [1]. In der Literatur werden vor allem eine homonyme Hemianopsie, migräneartige Kopfschmerzen, eine sensomotorische Halbseitensymptomatik sowie Sprachstörungen berichtet. Die Symptome sind meist nach wenigen Wochen vollständig oder unvollständig rückläufig [2]. Im Verlauf können weitere, vor allem klinisch ähnliche Episoden auftreten [3]. Diagnosekriterien für das SMART-Syndrom wurden 2006 von Black et al. verfasst und enthalten zu klinischen und bildgebenden Charakteristika auch den Ausschluss anderer möglicher Ursachen. Bislang konnte keiner Therapie ein Nutzen hinsichtlich einer rascheren Erholung oder Vorbeugung weiterer klinisch manifester Episoden nachgewiesen werden. In der Literatur werden jedoch häufig intravenöse Kortikosteroide in der Akutsituation eingesetzt, antithrombotische Therapien hingegen selten [2, 4].

Die pathophysiologische Ursache des SMART-Syndroms ist bislang nicht bekannt. Möglicherweise spielen jedoch strahleninduzierte Endotheldysfunktionen eine Rolle, die zu einer Veränderung der Blut-Hirn-Schranke und damit zu einer streng einseitigen kortikalen Ödembildung führen. Typische bildgebende Befunde zeigen, passend zu unserem Fall, eine vor allem okzipital und temporal betonte T2-Hyperintensität ohne relevante Diffusionsstörung [2, 5], wobei letzteres gelegentlich im Rahmen begleitender reversibler kortikaler Ischämien beobachtet werden konnte [6], so auch in unserem Fall. Auf Zellebene wird darüber hinaus eine Veränderung von Ionenkanälen angenommen, die wiederum ursächlich für epileptische Anfälle sein könnten. In der Literatur treten diese bei etwa 40 % der Patienten auf, in unserem Fall jedoch nicht [6]. Das EEG weist in der Literatur, ebenfalls passend zu unserem Befund, unspezifische Verlangsamungen, selten epilepsietypische Potenziale auf [3]. Eine Biopsie ist nicht zielführend und sollte als invasive Diagnostik lediglich unter dringendem bildgebendem Verdacht eines Tumorrezidivs herangezogen werden [1, 2]. Differenzialdiagnostisch sollte vor allem eine zerebrale Vaskulitis, infektiöse oder autoimmune Meningoenzephalitis, ein posteriores reversibles Enzephalopathiesyndrom (PRES) und ein postiktaler Zustand nach prolongierten epileptischen Anfällen in Betracht gezogen werden. Zur diagnostischen Abgrenzung erfolgte in unserem Fall, wie auch in den berichteten Fällen der Literatur, eine Liquoranalyse mit Bestimmung neurotroper Viren, antineuronaler und paraneoplastischer Antikörper sowie vaskulitisassoziierter Antikörper. Während sich bei der Meningoenzephalitis für gewöhnlich eine erhöhte Liquorzellzahl zeigt, ist dies beim SMART-Syndrom nicht der Fall. Auch Kerklaan und Singh et al. berichteten, passend zu unserem Befund, von einer diskret erhöhten Liquorproteinmenge bei normwertiger Zellzahl [7]. Spezifische antineuronale und paraneoplastische Antikörper sind nicht nachweisbar [6]. Die klinischen Manifestationen eines PRES und eines SMART-Syndroms können sich unter Umständen sehr ähnlich sein. Die Vorgeschichte einer zerebralen Bestrahlung lässt jedoch eher an das SMART-Syndrom denken, während Blutdruckunregelmäßigkeiten sowie autoimmune Erkrankungen für ein PRES sprechen. Bildgebend geht das PRES mit bilateralen T2-Hyperintensitäten des Marklagers und der kortikalen Region einher, was es von der einseitigen rein kortikalen Manifestation des SMART-Syndroms unterscheidet [8].

## Fazit für Praxis

- Bei Patienten mit der Vorgeschichte einer Hirnbestrahlung und unklaren fokal neurologischen Defiziten in Verbindung mit Kopfschmerzen sollte vor Einsatz invasiver Diagnostik an ein SMART-Syndrom gedacht werden.
- Typische bildgebende Befunde sind unilaterale, kortikale T2-Hyperintensitäten mit und ohne Diffusionsstörung.
- Therapien mit nachgewiesenem Nutzen existieren bislang nicht, hochdosierte Kortikosteroide können die Rückbildung der Symptome möglicherweise unterstützen.
